# Association between Cumulative BMI and Cognitive Decline: a 24-Year Cohort Study

**DOI:** 10.21203/rs.3.rs-6442210/v1

**Published:** 2025-05-20

**Authors:** Qianhui Xu, Meng Hsuan Sung, Zhuo Chen, Janani Rajbhandari-Thapa, Grace Bagwell Adams, M. Mahmud Khan, Ye Shen, Xiao Song, Xia Song, Suhang Song

## Abstract

Background: High Body Mass Index (BMI) is linked to poor cognitive performance, yet few studies have examined the long-term impact of cumulative BMI (cBMI) on cognitive health. This study explores the association between cBMI and cognitive decline and identifies the critical time window when cBMI has the strongest impact. Methods: Data were obtained from the Health and Retirement Study (1996–2020). Cognitive health was assessed using a standardized composite score of memory and executive function. Cumulative BMI was calculated as the area under the curve of BMI over time, and cumulative average BMI (caBMI) was computed as the mean of cBMI values over the follow-up period. Linear mixed models assessed the associations between caBMI and cognitive decline, adjusting for sociodemographic and health factors. Results: Among 8,252 cognitively healthy participants (mean age 58.6 years, 58.3% women, mean follow-up 17.5 years), a 100-unit increase in caBMI was significantly associated with faster cognitive decline: global cognition (-0.0030 SD/year, 95% CI: -0.0036, -0.0024), executive function (-0.0029 SD/year, 95% CI: -0.0038, -0.0021), and memory (-0.0017 SD/year, 95% CI: -0.0023, -0.0011) (all p < 0.001). Year eight was identified as the time point at which the association between caBMI showing the strongest decline rates in global cognition, memory, and executive function. Subgroup analyses revealed that caBMI was related to greater cognitive decline in older adults (≥ 65 years). Conclusions: caBMI was significantly associated with cognitive decline, with the largest impact observed eight years later. These findings highlight the importance of long-term weight management and BMI monitoring in cognitive health assessments.

### Introduction

Alzheimer's disease is the most prevalent form of dementia and has emerged as a leading cause of mortality in the US, with data indicating that 1 in 3 older adults died with Alzheimer's or another dementia in 2023 [1]. Currently, nearly 6.7 million US adults aged 65 or older are estimated to be living with dementia, and this number could rise to nearly 12 million by 2040 [2]. This alarming trend highlights a significant aging crisis, underscoring the urgent need for strategies to safeguard the cognitive health of the elderly. While no curative treatments for dementia currently exist, ongoing efforts to identify and adderess modifiable risk factors are crucial for preventing or delaying cognitive decline and the onset of dementia.

Higher Body Mass Index (BMI) has been reported to be one of the modifiable risk factors associated with poorer cognitive performance and faster cognitive decline [3]. Although the mechanisms remain unclear, one potential association may involve high BMI influencing brain structure and function through neuroinflammation and cerebrovascular dysregulation [4, 5]. These changes in critical brain regions have been associated with impairments in cognitive processes, including memory and executive function [6-8]. Additionally, high BMI may influence cognition by compromising neural factors essential for cognitive performance, such as short-chain fatty acids [9].

The role of longitudinal changes in BMI in cognitive decline remains unclear due to inconsistent evidence [4, 10-14]. While some studies suggest no significant association between BMI changes and cognitive decline, others report that individuals with a sustained high BMI at different time points tend to exhibit reduced cognitive performance later in life [15, 16]. These findings highlight the limitations of relying solely on single-point BMI measurements (e.g., baseline BMI or BMI at specific time points), or short-term BMI changes, as these approaches may fail to capture the dynamic and cumulative nature of BMI changes over time. Even studies examining BMI changes typically calculate them as the difference between two single time points, failing to account for the cumulative effect of BMI over extended periods [17, 18]. An alternative approach to relying on single-point or the difference in two single-point BMI measurements is to integrate BMI values across multiple time points, known as a cumulative BMI (cBMI). A cBMI approach offers a more comprehensive metric to assess the accumulated burden of high BMI over an extended period, which may better explain its role in cognitive decline. To date, no studies have identified the specific time periods during which BMI has the greatest effect on subsequent cognitive decline. A cumulative analysis allows for the identification of these critical windows, which is essential for understanding the relationship between BMI trajectory and cognitive function. This approach also provides valuable insights for health policies, guiding the development of targeted interventions to ensure that weight management strategies are implemented at the most effective time period.

To address existing gaps in empirical evidence, this study aimed to 1) investigate the longitudinal association between long-term cBMI and subsequent cognitive decline and 2) examine the critical time period during which cBMI significantly impacts cognitive decline.

## Methods

Data Source and Study Population

Data were obtained from a 24-year cohort Health and Retirement Study (HRS), spanning from Wave 3 (1996) to Wave 15 (2020). HRS is a nationally representative longitudinal study conducted biennially, mainly involving individuals aged 50 years and older[19]. Information regarding the objectives, design, and methodologies of the cohorts is available in other studies [20]. All HRS participants provided verbal informed consent for their participation in the study; HRS data collection procedures were approved by the National Institute on Aging and Institutional Review Board at the University of Michigan (HUM00061128) [19]. All variables used in the analysis are publicly available, and the analytic study was approved by the Institutional Review Board of the University of Georgia (approved IRB #PROJECT00008358).

This study began with a total sample of 40,130 participants from Wave 3 (1996). We excluded participants who (1) were missing baseline cognitive function measurements (n=29,905), (2) were diagnosed as cognitively impaired but not demented (CIND) or demented at baseline (n=1,327), (3) lacked data for BMI at baseline (n=123), (4) had only a single measurement for either BMI or cognitive function (n=520), (5) had missing values on covariates at baseline (n=3). The final analytic sample consisted of 8,252 participants. A flowchart detailing the selection process is in Figure 1 (Fig. 1).

#### Cumulative BMI Measurement

BMI was calculated by formula weight/height$^2$ using self-reported height (m) and weight (kg) [21]. cBMI was calculated using the Area Under the Curve (AUC) method, which plots BMI measurements over time [22, 23]. The trapezoidal rule was used to determine the area under the curve, representing long-term exposure to BMI levels [22, 24]. Cumulative average BMI (caBMI) was calculated as the mean of cBMI values recorded across the follow-up period in years for each participant, updated at each wave interval. Detailed information on the calculation of cBMI and caBMI was shown in the Supplementary Methods.

#### Assessment of Cognitive Function

The outcome variables included three cognitive measures: two cognitive domains (i.e., memory and executive function) and global cognition. Memory domain was evaluated via immediate word recall (score range: 0-10) and delayed word recall (range: 0-10) tests. Executive function was assessed using the combined score from serial 7 subtraction (range: 0-5) and backward counting (range: 0-2) tests [25, 26]. A global cognitive score (range: 0–27) was computed by summing scores from memory and executive function. Higher scores indicated better cognitive performance [25, 27]. Z-scores for each of the two cognitive domains and global cognition were calculated by subtracting the mean score at baseline from each individual data point in each wave and then dividing by the baseline standard deviation [28]. Cognitive decline rate was calculated as the change in standardized cognitive Z-scores from baseline to the follow-up period, divided by the total follow-up duration for each participant.

The analysis accounted for both time-fixed and time-varying covariates. Time-fixed covariates included demographic factors measured at baseline (1996): age, sex (men or women), race and ethnicity (non-Hispanic White, non-Hispanic Black, non-Hispanic Other, or Hispanic), and educational attainment (less than high school, GED, high school graduate, college, or college and above). Time-varying covariates included depression status (yes or no), smoking status (never smoked, ever smoked, or currently smoking), insurance status (insured or uninsured), employment status (employed, unemployed, retired, disabled, or not in the labor force), and the number of chronic diseases. The number of chronic diseases, ranging from 0 to 6, represents the summation of binary indicators for the following conditions: 1) hypertension or high blood pressure, 2) high blood sugar or diabetes, 3) cancer or any malignant tumor excluding skin cancer, 4) chronic lung disease excluding asthma, such as chronic bronchitis or emphysema, 5) heart attack, coronary heart disease, congestive heart failure, angina, or other heart conditions, and 6) transient ischemic attack or stroke.

#### Statistical Analysis

We conducted descriptive statistical analyses to summarize baseline characteristics. Continuous variables were reported as means with standard deviations (SD), while categorical variables were presented as frequencies and percentages. Statistical significance was set at the 5% level ($p<0.05$) with two-tailed tests. All analyses were conducted using R Studio (Version 2024.04).

To examine the longitudinal association between caBMI and cognitive decline rate, we applied linear mixed models which incorporated random intercepts at the participant level to account for baseline differences and individual variations. The models included an interaction term of cumulative average BMI (caBMI) and time, where time was measured as the number of years elapsed since the baseline year (1996) when cBMI was calculated, with a negative coefficient of the interaction term interpreted as indicative of a faster decline rate in cognition while holding time constant [29, 30].

We employed three models to sequentially add sets of characteristics, accounting for both nonmodifiable and modifiable factors. Model 1 regressed caBMI on cognitive decline rate adjusted for demographic characteristics including age, gender, and race/ethnicity to account for nonmodifiable characteristics; Model 2 additionally adjusted for socioeconomic status (SES) (i.e., educational attainment, insurance, and labor status) to reflect modifiable factors; and Model 3 was a fully adjusted model with additional encompassing health conditions (i.e., smoking, depression, number of chronic diseases).

To determine the time period during which caBMI has the greatest impact on cognitive decline, we applied a linear mixed model to analyze lag years across intervals ranging from 2 to 16 years. Lag years refer to the temporal delay between the measurement of the caBMI and its subsequent impact on the rate of cognitive decline. For example, lag year two refers to the time interval where the cognitive decline is assessed two years after the caBMI's measurement. To better understand the influence of caBMI on cognitive decline across population groups, we further conducted stratification analyses by age, sex, race and ethnicity, depression status, educational attainment, and insurance groups. We also performed a sensitivity analysis in which we excluded participants with chronic diseases at baseline and examined the association between caBMI and cognitive decline.

### Results

#### Study Population Characteristics

Table 1 presents the participants' baseline characteristics. At baseline, the study sample had an average age of 58.6 years (SD=5.6), with women comprising 58.3% of the cohort. The racial/ethnic composition was predominantly non-Hispanic White (78.7%). Most participants were either retired (33.9%) or not in the labor force (52.2%). Additionally, 39.5% of participants reported never smoking, while 39.5% had smoked in the past. The baseline mean score on global cognition was 17.7(SD=3.3), with a mean memory score of 11.8 (SD=3.0) and a mean executive function score of 5.9 (SD=1.4). The follow-up duration was 17.5 (SD=7.0) years on average. The mean BMI is 27.3(SD=5.1), while the mean caBMI is 27.7(SD=5.1)

#### Association between caBMI and cognitive decline

Table 2 presents the results of progressively adjusted models evaluating the relationship between caBMI and global cognitive decline rate, using an 8-year lag as an example. The magnitude of the relationship between caBMI and cognitive decline was consistent across all three models, sequentially adjusting for nonmodifiable and modifiable factors (Table 2). Specifically, an increase of 100-unit cumulative average BMI was related to an accelerated decline rate of 0.0030 SD/year in global cognition (0.0030; 95% CI: -0.0036, -0.0024; p<0.001). A statistically significant association was observed in declines in memory and executive function, with a faster decline rate of 0.0017 SD/year (-0.0017; 95% CI: -0.0023, -0.0011; p<0.001) and 0.0028 SD/year (0.0028; 95% CI: -0.0034, -0.0021; p<0.001) with an increase in 100-unit cumulative average BMI, respectively (Supplementary Table 2 and 3).

In the sensitivity analyses, in which individuals with chronic diseases at baseline were excluded, the results remained consistent, thus demonstrating no substantial changes in the association between higher caBMI and accelerated cognitive decline rate (Supplementary Table 4).

#### Identification of the critical period

Table 3 presents the magnitude of the association between caBMI and subsequent cognitive decline rates over the study period. Lag year eight was identified as the critical period during which caBMI showed the greatest association with cognitive decline rate. To illustrate, global cognition showed consistent and significant decline rates over time, peaking at year 8 (-0.0030; 95% CI: -0.0036, -0.0024; p<0.001). Decline rates of global cognition accelerated from -0.0008 SD/year at 2 years (-0.0008; 95% CI: -0.0016, -0.0002; p=0.015) to -0.0022 SD/year at 4 years (-0.0022; 95% CI: -0.0028, -0.0016; p<0.001), and -0.0018 SD/year at 6 years (-0.0018; 95% CI: -0.0024, -0.0012; p<0.001). Then, after peaking at year 8, the decline rates gradually stabilized, reducing to -0.0028 SD/year at 10 years (-0.0028; 95% CI: -0.0034, -0.0021; p<0.001) to -0.0016 SD/year at 16 years (-0.0016 95% CI: -0.0030, -0.0004; p=0.010). At year 18, the association was no longer statistically significant (-0.0002; 95% CI: -0.0020, 0.0016; p=0.825), likely due to the smaller sample size which leads to less stable effect estimates. Executive function and memory exhibited a similar trajectory to global cognition, with significant negative effects of caBMI observed consistently across most lag years, spanning from year 2 to year 16. The decline rates were most pronounced at year 8 for both domains, consistent with the findings for global cognition. Across all three cognitive assessments, lag year 8 consistently emerged as a critical time point in the association between caBMI and cognitive decline, where weight management strategies could be most effective in slowing cognitive decline.

#### Subgroup analyses

After stratifying participants into subgroups, this study revealed statistically significant negative effects of caBMI on global cognition rate across each group by age, gender, and educational attainment. The results are presented in Figure 2 (Fig. 2). Among participants aged ≥65 years, an increase of 100-unit cumulative average BMI was linked to an accelerated decline rate of 0.0075 SD/year (95% CI: -0.0096, -0.0053; $p < 0.001$) for global cognition. This negative relationship was significantly stronger than the participants younger than 65 years (-0.0020 SD/year; 95% CI: -0.0026, -0.0014; $p<0.001$). Besides, the effects of increased caBMI on global cognitive decline were relatively consistent across gender and educational attainment, with similar decline rates observed for both men and women, as well as across all educational attainments. Similar trends were also observed in memory and executive function domains, highlighting the greater susceptibility of older adults to the impact of caBMI on cognitive decline.

### Discussion

This population-based cohort study with average cBMI of 27.7 (SD = 5.2) over a follow-up period of 17.5 years (SD = 7.0) innovatively demonstrated that higher cBMI exposure is associated with subsequent cognitive decline, with the greatest association being observed in the following eighth year after the initial measurement of caBMI. By measuring longitudinal BMI exposure, our findings highlight the importance of sustained BMI management and its role in long-term prevention strategies for cognitive decline. The link between higher cumulative BMI (cBMI) and faster declines in global cognition, memory, and executive function suggests that long-term BMI patterns should be considered in cognitive risk assessments by clinicians.

As a modifiable risk factor, BMI represents a crucial target for interventions designed to reduce obesity-related cognitive decline, although some studies observed that accumulation of cognitive reserve may help buffer against the negative effects of high BMI on cognitive function in later life [31, 32]. Our study aligns with previous research demonstrating that higher BMI is associated with longitudinal neurocognitive impairment [33] and cross-sectionally poorer cognitive function [34], further reinforcing the association between elevated BMI and an accelerated decline in cognitive performance. The biological mechanism between BMI and cognitive health remains unclear, but prior research has proposed several pathways linking high BMI to greater cognitive decline [5, 35, 36]. Notable mechanisms include systemic inflammation leading to neuroinflammation, impairments in cerebral circulation that disrupt the ability to match blood flow to the metabolic demands of neurons, and cerebrovascular dysregulation characterized by reduced functional hyperemia and endothelial dilation [5, 36, 37]. Additionally, some studies observed long-term high BMI may be associated with alterations in gut microbiota, leading to reduced short-chain fatty acid production, which impacts brain function by modifying microglial activity and disrupting gene expression for neurons [38, 39]. Furthermore, our findings highlight the association of cBMI with memory and executive function, offering a more specific understanding of the cBMI-cognitive decline relationship [40, 41]. Previous research has indicated that high BMI is associated with impaired hippocampal vascular health and structural changes in regions involved in memory functions, such as the angular gyrus, which induce declines in memory processing and retrieval [42, 43]. On the other hand, executive function is regulated by the prefrontal cortex, which is responsible for complex behaviors such as decision-making and reasoning [44]. Gray matter volume and blood flow in the prefrontal cortex may reduce as BMI increases, which may help explain the negative correlation between cBMI and executive function [45, 46].

In this study, we innovatively identified year 8 as a critical period when the cBMI had the strongest potential impact on cognitive decline. This time frame aligns with prior research on BMI trajectories, which found that changes in BMI are linked to cognitive decline approximately seven years later [47]. Our findings highlight this time window as a valuable reference point for future research and shed light on BMI management during this critical period. Besides, subgroup analyses further reveal an increased susceptibility to the association between cBMI and cognitive decline, particularly among older adults. This observation aligns with previous studies that identify aging populations as more vulnerable to high BMI-related cognitive decline [48, 49]. Our finding emphasizes the need for targeted interventions aimed at weight management in older populations to reduce the risk of accelerated cognitive decline.

From a clinical perspective, the results of cBMI underscore the importance of longitudinal BMI monitoring and sustained weight management interventions to mitigate the cumulative impact of BMI on cognitive function. Furthermore, the identification of an 8-year critical window emphasizes the need for timely interventions during this pivotal period to optimize cognitive health outcomes. From a policy perspective, our findings on cBMI emphasize the need for public health policies that prioritize strategies addressing the cumulative burden of obesity. These strategies should focus on promoting sustained lifestyle changes to maintain a healthy BMI. Based on the finding that the eighth year is the most critical window of time, we further suggest policy efforts could focus on developing targeted intervention programs tailored to the identified 8-year window, maximizing the effectiveness of prevention strategies for cognitive decline.

This study includes several limitations. First, the reliance on self-reported BMI may introduce recall bias, and social desirability bias, which potentially affects the accuracy of the measurement. Second, the presence of missing data constrains the generalizability of our findings, which are primarily applicable to individuals with characteristics similar to those in the study population. Third, other covariates may not have been accounted for due to the availability of the data, such as environmental exposures (e.g., local pollutions), which might also impact the relationship between cBMI and cognitive decline.

Our study has several notable strengths. First, we innovatively capture the long-term effects of BMI, offering a more nuanced understanding of its relationship with cognitive outcomes, compared with traditional single-point BMI measurements. Second, our analysis identifies year 8 as a critical period when the impact of BMI on cognitive decline is most significant, highlighting critical time windows for implementing targeted interventions to mitigate cognitive decline. Third, the 24-year longitudinal design enables a comprehensive analysis of BMI trajectories and their association with cognitive decline, offering valuable insights into long-term trends.

## Conclusions

The study highlights the significant association between greater cBMI and accelerated declines in global cognition, memory, and executive function. Importantly, the identification of year 8 as a critical period underscores the importance of implementing targeted interventions during this key time to decrease the risk of long-term cognitive decline. Future investigation is warranted to investigate the biological mechanisms underlying the association between cBMI and cognitive decline and develop targeted interventions focused on this critical time window to enhance cognitive health.

## Declarations

**Funding Declaration:** This work was supported by the National Center for Advancing Translational Sciences of the National Institutes of Health under Award Number UL1TR002378. The content is solely the responsibility of the authors and does not necessarily represent the official views of the National Institutes of Health.

**Human Ethics and Consent to Participate Declarations**: not applicable

**Clinical Trial Number**: not applicable

**Consent for Publication Declarations:** not applicable

**Competing Interests**: The authors declare that they have no known competing financial interests or personal relationships that could have appeared to influence the work reported in this paper.

## Tables

**Table 1 Baseline characteristics of participants**

| Variables | Characteristics |
|---|---|
| Global Cognition (mean [SD]) | 17.7 (3.3) |
| Memory (mean [SD]) | 11.8 (2.9) |
| Executive Function (mean [SD]) | 5.9 (1.4) |
| Age (mean [SD] in years) | 58.5 (5.5) |
| Sex (%) | |
| Men | 3446 (41.7) |
| Women | 4806 (58.2) |
| Race/Ethnicity (%) | |
| Non-Hispanic White | 6492 (78.6) |
| Non-Hispanic Black | 1024 (12.4) |
| Non-Hispanic Other | 146 (1.7) |
| Hispanic | 590 (7.1) |
| Educational Attainment (%) | |
| Lower Than High-school | 1548 (18.7) |
| GED | 418 (5.0) |
| High-school graduate | 2817 (34.1) |
| Some College | 1796 (21.7) |
| College and above | 1673 (20.2) |
| Labor Status (%) | |
| Employed | 726 (8.8) |
| Unemployed | 166 (2.0) |
| Retired | 2796 (33.8) |
| Disabled | 258 (3.1) |
| Not in labor force | 4306 (52.1) |
| Insurance (%) | |
| Yes | 7436 (90.5) |
| No | 773 (9.4) |
| Smoking (%) | |

| | |
|---|---|
| Never smoked | 3111 (39.4) |
| Ever smoked | 3117 (39.5) |
| Current smoking | 1659 (21.0) |
| Depression (%) | |
| Yes | 7254 (87.9) |
| No | 995 (12.0) |
| BMI (mean [SD]) | 27.3 (5.1) |
| Number of Chronic Diseases (mean [SD]) | 0.8 (0.9) |
| Follow Up Duration Years (mean [SD]) | 17.5 (7.0) |

Abbreviations: SD: standard deviation; BMI: Body Mass Index; GED: General Educational Development.

**Table 2 The association between cumulative average BMI and global cognitive decline**

| | Model 1 | | Model 2 | | Model 3 | |
|---|---|---|---|---|---|---|
| | Coefficient 95% CI | P-value | Coefficient 95% CI | P-value | Coefficient 95% CI | P-value |
| **caBMI** | -0.0021 -0.0366, 0.0323 | 0.904 | 0.0113 -0.0232, 0.0459 | 0.521 | 0.0142 -0.0209, 0.0494 | 0.428 |
| **caBMI*Time** | -0.0030 -0.0035, -0.0025 | <0.001 | -0.0032 -0.0038, -0.0026 | <0.001 | -0.0030 -0.0036, -0.0024 | <0.001 |
| **Race/Ethnicity** | | | | | | |
| **Non-Hispanic White** | ref | | ref | | ref | |
| **Non-Hispanic Black** | -0.0240 -0.0295, -0.0184 | <0.001 | -0.0216 -0.0272, -0.0161 | <0.001 | -0.0212 -0.0268, -0.0156 | <0.001 |
| **Non-Hispanic Other** | -0.0138 -0.0267, -0.0008 | 0.037 | -0.0134 -0.0264, -0.0005 | 0.040 | -0.0112 -0.0242, 0.0018 | 0.091 |
| **Hispanic** | -0.0101 -0.0170, -0.0032 | 0.004 | -0.0021 -0.0093, 0.0050 | 0.562 | -0.0020 -0.0092, 0.0053 | 0.598 |
| **Age** | -0.0029 -0.0033, -0.0026 | <0.001 | -0.0029 -0.0032, -0.0025 | <0.001 | -0.0029 -0.0033, -0.0026 | <0.001 |
| **Sex** | | | | | | |
| **Men** | ref | | ref | | ref | |
| **Women** | -0.0038 -0.0075,0.00004 | 0.048 | -0.0018 -0.0056, 0.0020 | 0.349 | -0.0029 -0.0067, 0.0010 | 0.150 |
| **Educational Attainment** | | | | | | |
| **Lower Than High-School** | | | ref | | ref | |
| **GED** | | | 0.0124 0.0035, 0.0214 | 0.007 | 0.0127 0.0037, 0.0217 | 0.006 |
| **High-School Graduate** | | | 0.0139 0.0086, 0.0193 | <0.001 | 0.0127 0.0073, 0.0181 | <0.001 |
| **Some College** | | | 0.0159 0.0101, | <0.001 | 0.0154 0.0095, | <0.001 |

| | | | | |
|---|---|---|---|---|
| | 0.0217 | | 0.0213 | |
| **College and above** | 0.0212<br>0.0153,<br>0.0271 | <0.001 | 0.0190<br>0.0130,<br>0.0251 | <0.001 |
| **Labor Status** | | | | |
| **Not in labor force** | ref | | ref | |
| **Employed** | 0.0043<br>0.0008,<br>0.0079 | 0.015 | 0.0044<br>0.0008,<br>0.0080 | 0.016 |
| **Unemployed** | 0.0074<br>0.0007,<br>0.0141 | 0.030 | 0.0080<br>0.0013,<br>0.0148 | 0.020 |
| **Retired** | 0.0038<br>0.0006,<br>0.0071 | 0.021 | 0.0046<br>0.0013,<br>0.0079 | 0.007 |
| **Disabled** | -0.0034<br>-0.0097,<br>0.0029 | 0.290 | -0.0013<br>-0.0078,<br>0.0053 | 0.708 |
| **Insurance** | | | | |
| **No** | ref | | ref | ref |
| **Yes** | 0.0073<br>0.0036,<br>0.0110 | 0.000 | 0.0072<br>0.0035,<br>0.0110 | 0.000 |
| **Smoking** | | | | |
| **Never smoked** | | | ref | ref |
| **Ever smoked** | | | -0.0026<br>-0.0064,<br>0.0012 | 0.174 |
| **Current smoking** | | | -0.0048<br>-0.0094,<br>-0.0003 | 0.037 |
| **Depression** | | | | |
| **No** | | | ref | |
| **Yes** | | | -0.0026<br>-0.0050,<br>-0.0002 | 0.033 |
| **Number of Chronic Diseases** | | | -0.0018<br>-0.0029,<br>-0.0007 | 0.002 |

Note. The caBMI was measured in 100-unit scale.

Abbreviations: SD: standard deviation; BMI: Body Mass Index; GED: General Educational Developme

**Table 3 The association between cumulative average BMI and subsequent cognitive decline in the following 2 to 18 years**

| | Global Cognition<br>Coeff.<br>95%CI<br>p-value | Memory<br>Coeff.<br>95%CI<br>p-value | Executive Function<br>Coeff.<br>95%CI<br>p-value |
|---|---|---|---|
| **2 years** | -0.0009<br>-0.0016 - 0.0002<br>0.015 | -0.0006<br>-0.0013, 0.0001<br>0.113 | -0.0005<br>-0.0012, 0.0002<br>0.149 |
| **4 years** | -0.0022<br>-0.0028, -0.0016<br><0.001 | -0.0013<br>-0.0019, -0.0006<br>0.001 | -0.0020<br>-0.0026, -0.0014<br><0.001 |
| **6 years** | -0.0018<br>-0.0024, -0.0012<br><0.001 | -0.0008<br>-0.0014, -0.0002<br>0.014 | -0.0021<br>-0.0027, -0.0015<br><0.001 |
| **8 years** | -0.0030<br>-0.0036, -0.0024<br><0.001 | -0.0017<br>-0.0023, -0.0011<br><0.001 | -0.0028<br>-0.0034, -0.0021<br><0.001 |
| **10 years** | -0.0028<br>-0.0034, -0.0021<br><0.001 | -0.0016<br>-0.0022, -0.0009<br><0.001 | -0.0022<br>-0.0028, -0.0015<br><0.001 |
| **12 years** | -0.0014<br>-0.0022, -0.0007<br><0.001 | -0.0005<br>-0.0013, 0.0002<br>0.168 | -0.0009<br>-0.0016, -0.0002<br>0.019 |
| **14 years** | -0.0012<br>-0.0022, -0.0003<br>0.009 | -0.0003<br>-0.0013, 0.0006<br>0.465 | -0.0004<br>-0.0013, 0.0005<br>0.353 |
| **16 years** | -0.0016<br>-0.0028, -0.0004<br>0.010 | 0.0003<br>-0.0009, 0.0015<br>0.640 | -0.0018<br>-0.0030, -0.0006<br>0.003 |

## Figures

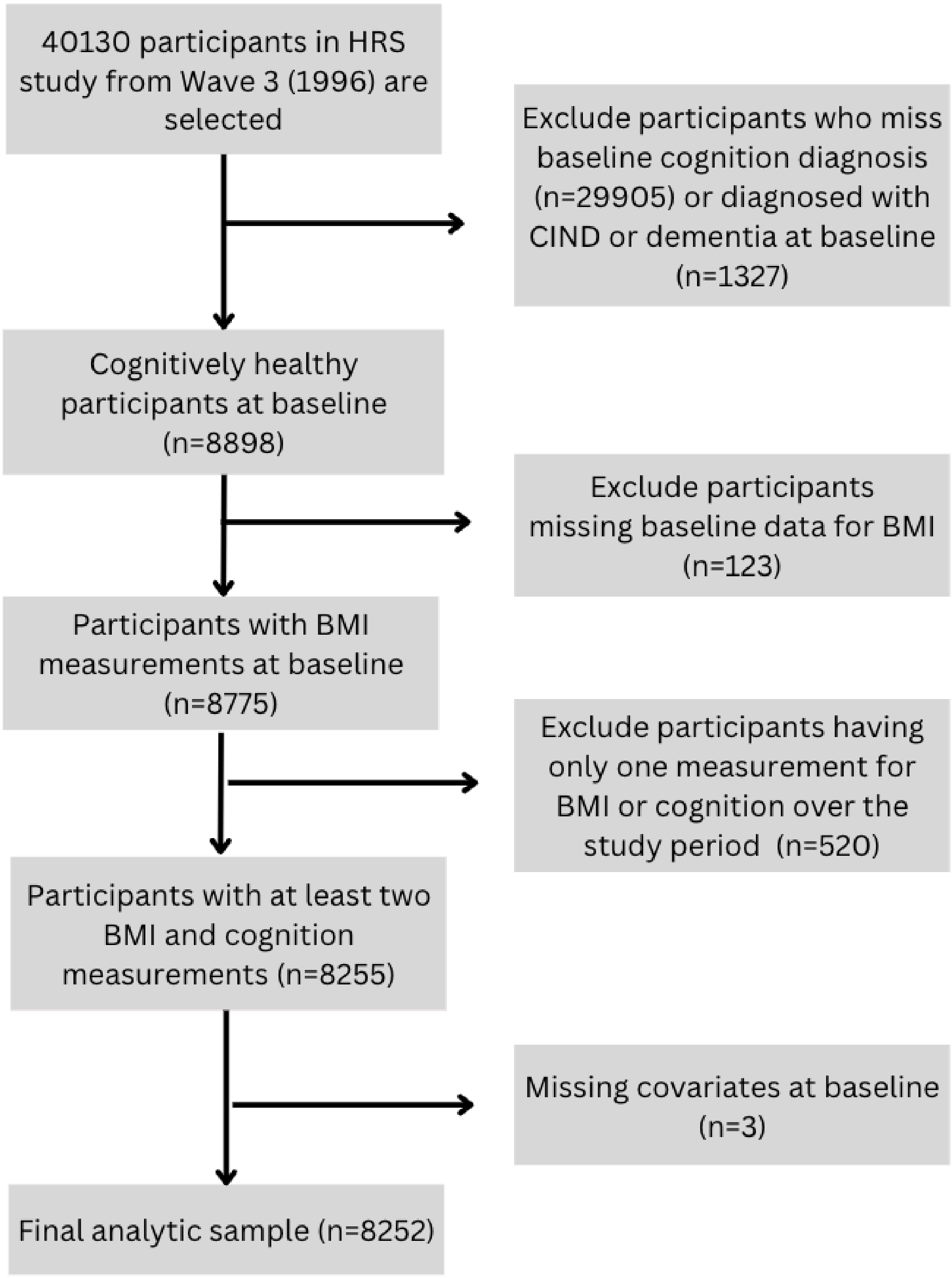

**Figure 1**

Flowchart of participants selection

Abbreviation: HRS: Health and Retirement Study; CIND: Cognitive impairment with no dementia; BMI: Body Mass Index

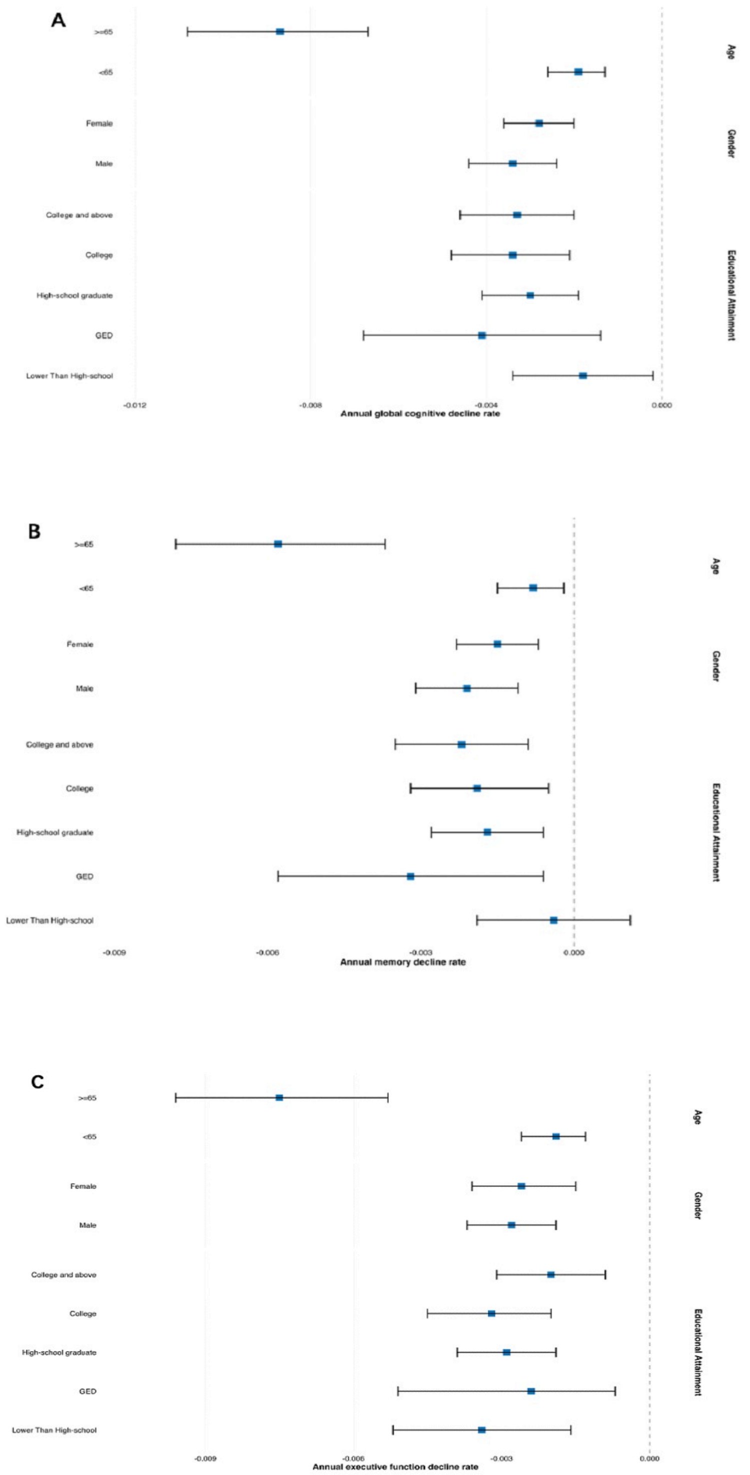

### Figure 2

The association of cumulative average BMI with declines in global cognition (A), memory (B), and executive function (C) by age, gender, and educational attainment

## Supplementary Files

This is a list of supplementary files associated with this preprint. Click to download.

- SupplementalMaterialDPH.pdf
